# Initial Performance of a Prototype Small Animal PET Scanner Consisting of Long Semi-Monolithic Scintillator Detectors

**DOI:** 10.3390/s26154943

**Published:** 2026-08-05

**Authors:** Zhonghua Kuang, Samuel Mungai Kinyanjui, Ling Zhang, Ning Ren, Haonan Li, Zhanli Hu, Yongfeng Yang, Zheng Liu

**Affiliations:** 1 Microelectronics and Optoelectronics Technology Key Laboratory of Hunan Higher Education, School of Physics and Electronic Electrical Engineering, Xiangnan University, Chenzhou 423000, China; zhangl202107@163.com; 2 Paul C. Lauterbur Research Center for Biomedical Imaging, Shenzhen Institutes of Advanced Technology, Chinese Academy of Sciences, Shenzhen 518055, China; kinyanjui@siat.ac.cn (S.M.K.); ning.ren@siat.ac.cn (N.R.); hn.li@siat.ac.cn (H.L.); zl.hu@siat.ac.cn (Z.H.); yf.yang@siat.ac.cn (Y.Y.); 3 State Key Laboratory of Biomedical Imaging Science and System, Shenzhen Institutes of Advanced Technology, Chinese Academy of Sciences, Shenzhen 518055, China

**Keywords:** semi-monolithic detector, depth of interaction (DOI), small animal PET, high spatial resolution, GATE simulation

## Abstract

Semi-monolithic scintillator detectors possess not only the inherent depth of interaction (DOI) encoding capability of monolithic scintillator detectors but also reduced edge effects compared to monolithic scintillator detectors. The performance of a small animal PET scanner consisting of long semi-monolithic scintillator detectors was investigated in this work. Two semi-monolithic scintillator detectors composed of 12 LYSO slabs of 0.96 × 56 × 10 mm^3^ with ESR or BaSO_4_ as the reflectors were manufactured. First, a prototype small animal PET scanner consisting of the two semi-monolithic detectors and a rotation platform was built. The spatial resolution of the prototype PET scanner was measured using a ^22^Na point source. Second, the sensitivity and imaging performance of a full-ring small animal PET scanner consisting of 20 semi-monolithic detectors was simulated using GATE software. The proposed position calibration method for semi-monolithic scintillator detectors effectively addressed non-uniform position responses in both the monolithic and DOI directions and was suitable for system-level PET scanner calibration. Using experimental data from the two-detector prototype PET scanner, spatial resolution better than 0.65 mm was obtained with point spread function (PSF) modelling. In Monte Carlo simulations of a full-ring PET scanner consisting of 20 such detectors, a peak sensitivity of 5.45% was achieved, and rods with a diameter of 0.75 mm were clearly resolved in phantom images reconstructed with PSF modelling. Long semi-monolithic scintillator detector represents a promising candidate for developing small animal PET systems to achieve sub-millimeter uniform spatial resolution, good sensitivity, and reduced cost.

## 1. Introduction

Small animal positron emission tomography (PET) is a non-invasive molecular imaging tool widely used in biomedical research [1,2,3]. Small animal PET scanners need a high spatial resolution to minimize the partial volume effect so as to achieve high quantitative accuracy in small lesion detection [3,4]. Depth of interaction (DOI) encoding detectors are essential for small animal PET scanners to simultaneously achieve high spatial resolution and high sensitivity by reducing the parallax error [5,6,7].

**Figure 1:**
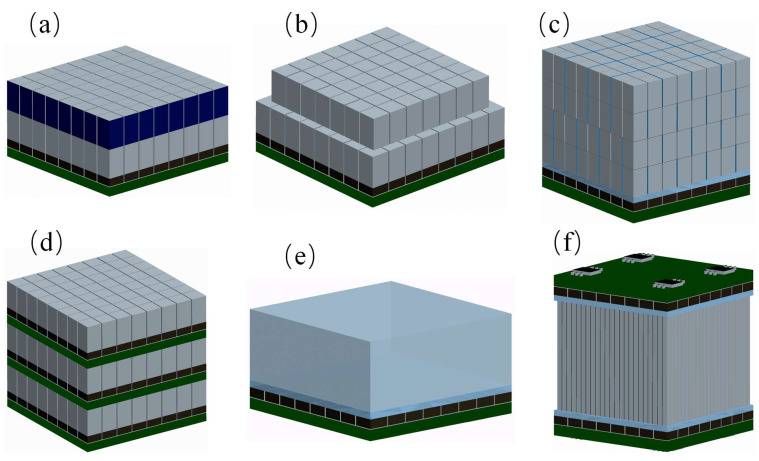
Schematics of DOI-encoding PET detectors. (**a**) Phoswich detector, (**b**) crystal arrays with multiple offset layers, (**c**) multiple crystal layers with stair-shaped reflector arrangement, (**d**) multiple individual single-ended readout detector layers, (**e**) monolithic scintillators and (**f**) dual-ended readout of segmented crystal arrays.

A few common designs of DOI-encoding PET detectors are illustrated in Figure 1. These include dual-ended readout of segmented crystal arrays [8,9], crystal arrays with multiple offset crystal layers [10,11], multiple crystal layers with stair-shaped reflector arrangement [12], monolithic scintillators [13,14], single-ended readout crystal arrays based on light sharing [15], and phoswich detectors that utilize multiple crystal layers with different scintillation decay times [5,16].

Most currently developed small-animal PET scanners that use detectors with DOI measurement capability adopt either dual-ended readout of segmented crystal arrays [17,18], monolithic scintillators [14,19,20] or single-ended readout of multi-layer crystal arrays [5,6,21,22,23]. Semi-monolithic scintillator detectors consisting of scintillator slabs with single-ended photosensor readout cannot only maintain the same inherent DOI-encoding capability as monolithic scintillator detectors, but also significantly reduce edge effects compared with those [24,25]. Compared with dual-ended readout detectors, semi-monolithic scintillator detectors require only one photosensor per module, which results in lower system costs. Furthermore, the edge effects in semi-monolithic detectors can be further reduced by increasing the scintillator slab length, which enables the development of small animal PET scanners with uniform high spatial resolution, high sensitivity, and low cost [26,27].

Silicon photomultipliers (SiPMs) have emerged as the photosensor of choice for modern PET and radiation detection systems, replacing the traditional photomultiplier tubes (PMTs). As solid-state photon sensors, SiPMs offer distinct advantages, including high photon detection efficiency (PDE), compact size, low operating voltage, and insensitivity to magnetic fields, making them ideal for multimodal imaging systems like PET/CT, PET/MRI, and SPECT/CT [28]. The development of high-resolution PET detectors relies heavily on the performance of the SiPM sensor.

The concept of the semi-monolithic PET detector was first proposed by Chung from Yonsei University in 2008, accompanied by preliminary simulation studies [24,25]. In recent years, semi-monolithic PET detectors have attracted increasing attention, with extensive research conducted on detector parameter optimization, positioning algorithm development, and detector performance evaluation [26,27,29,30,31,32,33,34]. However, only one small-animal PET scanner based on semi-monolithic detectors with scintillator slabs of 0.97 × 25.6 × 12 mm^3^ was developed in 2025. The scanner achieved an in-plane spatial resolution of less than 1 mm, but the axial resolution was poorer than 2.4 mm at the center of the field of view (FOV) [35]. In our previous work, semi-monolithic scintillator detectors composed of lutetium yttrium oxyorthosilicate (LYSO) slabs of 0.96 × 56 × 10 mm^3^ were developed and evaluated [27]. The two semi-monolithic detectors using ESR and BaSO_4_ reflectors provided similar y resolutions of ~1.7 mm and DOI resolutions of ~2.1 mm. The longer semi-monolithic scintillator detector design significantly reduced edge effects while maintaining inherent DOI-encoding capability and had the potential for whole-body mouse imaging. In this work, the spatial resolutions of a prototype small PET scanner consisting of those two semi-monolithic scintillator detectors were measured experimentally. The sensitivity and imaging performance of a small animal PET scanner with 20 semi-monolithic scintillator detectors of identical parameters were simulated.

## 2. Materials and Methods

### 2.1. Detectors

**Figure 2:**
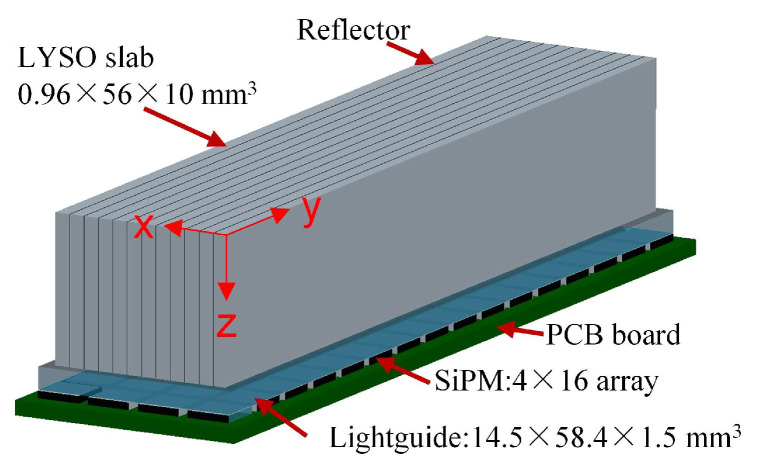
Schematic of a semi-monolithic detector module.

The schematic of a semi-monolithic scintillator detector module is shown in Figure 2. The semi-monolithic scintillator array consisted of 12 LYSO slabs of 0.96 × 56 × 10 mm^3^ (Epic Crystal Co. Ltd., Shanghai, China). The front and both end faces of the scintillator slabs were left unpolished and coated with black paint, whereas the two large lateral surfaces together with the bottom surface—destined for coupling to the SiPM sensor array—underwent optical polishing. The overall dimensions of the semi-monolithic crystal array was 12.4 × 56 × 10 mm^3^. The enhanced specular reflector film (ESR) and BaSO_4_ were used as the reflectors of the two detectors used in this work, respectively. Comprehensive performance data for both semi-monolithic detectors have been reported in our previous publication [27].

We employed a custom-fabricated 4 × 16 array of 64 Hamamatsu S14160-3050HS SiPM (Hamamatsu Photonics K.K, Tokyo, Japan) sensors to read out each semi-monolithic scintillator array. This photosensor featured a pixel pitch of 3.65 mm and an active area of 3 × 3 mm^2^ per channel. A light guide (14.5 × 58.4 × 1.5 mm^3^ in size) featuring two grooves (0.2 mm in width and 1 mm in depth) located 1 mm from the edges was used [36,37]. Optical grease was applied to both interfaces: scintillator-to-light guide and light guide-to-SiPM. Signal reduction from 64 to 20 channels (4 rows + 16 columns) was realized through a dedicated row-column multiplexing circuit. The 4 row signals were summed to generate the timing signal by the leading-edge discrimination method through a high-speed comparator with a threshold of 50 mV. The 40 output signals from the two semi-monolithic detectors were processed by a 64-channel singles processing unit previously developed for the SIAT bPET scanner (Shenzhen Institutes of Advanced Technology, Chinese Academy of Sciences, Shenzhen, China) [38,39] and saved as list-mode data. The center of gravity (COG) of the 4 row signals was used for slab identification (x-direction). The y-position was measured using the squared COG of the 16 column signals. The DOI (z-direction) was measured using the inverse standard deviation of the 16 column signals [27]. The x, y, and DOI positions were calculated using the following equations:

(1) $$x=\frac{\sum_{i=1}^{N}i\times e_{i}}{(N+1)\sum_{i=1}^{N}e_{i}}$$

(2) $$y=\frac{\sum_{j=1}^{M}j\times e_{j}^{2}}{(M+1)\sum_{j=1}^{M}e_{j}^{2}}$$

(3) $$DOI=\frac{1}{\sqrt{\frac{1}{M}\sum_{j=1}^{M}{\left({e_{n}}_{j}-\bar{e_{n}}\right)}^{2}}}$$

where i and j are the row and column index of the SiPM signals, with N = 4 and M = 16 being the total numbers of rows and columns, respectively. $e_{i}$ is the energy of the i-th row signal, and $e_{j}$ is the energy of the j-th column signal. ${e_{n}}_{j}$ is the normalized energy of the j-th column signal, and $\bar{e_{n}}$ is the average energy of the 16 column signals [27].

### 2.2. Experimental Measurements of a Prototype PET Scanner Consisting of Two Detectors

**Figure 3:**
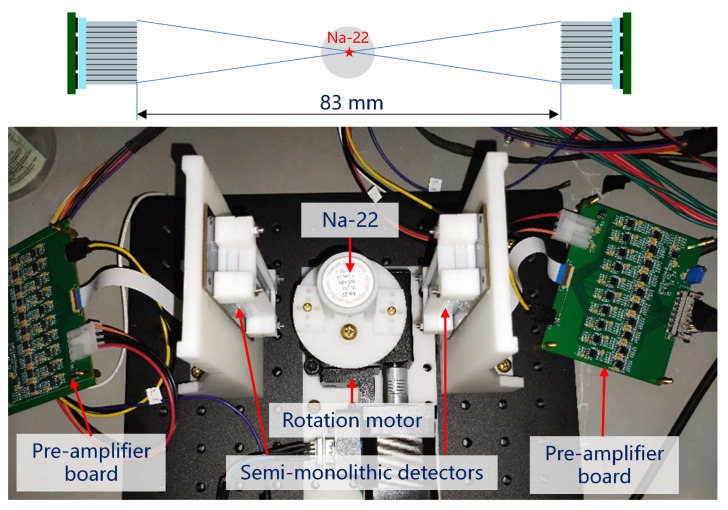
(**top**) Schematic and (**bottom**) photograph of the prototype PET scanner consisting of two semi-monolithic detectors and rotation platform.

**Figure 4:**
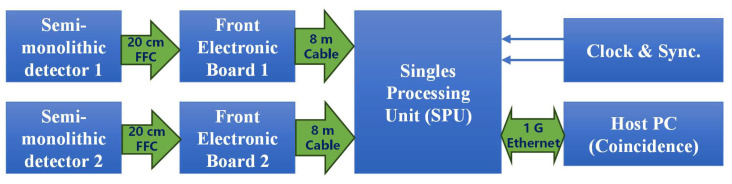
The electronics diagram of the prototype small animal PET scanner.

A schematic and a photograph of the prototype small animal PET scanner consisting of two semi-monolithic scintillator detectors and a rotation platform are shown in Figure 3. The electronics diagram of the prototype small animal PET scanner is shown in Figure 4. The distance between the front faces of the two detectors was 83 mm. First, the detector calibration data were acquired using the ^176^Lu background in the LYSO scintillator for 30 min. Then, detector position lookup tables (LUTs) for both the y and DOI directions were obtained by assuming that the number of events of the ^176^Lu background was uniform in the detector volume [40]. Next, a 0.3 mm diameter ^22^Na point source with an activity of 128 kBq was placed on the rotation platform. The spatial resolution of the prototype PET scanner was evaluated by placing the point source at radial offsets of 0 and 5 mm. For each position, the point source was measured for 10 angles with an interval of 18 degrees. The data acquisition time at each angle was 120 s. A sinogram of the point source was generated from all data of the 10 angles of each position. The images were reconstructed using a sinogram-based ordered-subsets expectation maximization (OSEM) algorithm with point spread function (PSF) modeling [41]. The dimensions of the sinogram were 150 (nDistance) × 128 (nView) × 56 (nRing) × 56 (nRing). The image dimensions were 150 × 150 × 111, and the voxel size is 0.4 × 0.4 × 0.5 mm^3^. The PSF parameters were obtained from the image of the point source placed at the center and reconstructed without PSF modeling. Line profiles passing through the brightest voxel of the point source images in three dimensions were fitted with Gaussian functions, and the full width at half maximum (FWHM) values of the profiles were used to represent the spatial resolution. The normalization correction data were obtained from Monte Carlo simulation of a uniform cylinder phantom, and direct normalization was applied during image reconstruction. A coincidence timing window of 20 ns was used during the data processing, and an energy window of 350–650 keV was applied.

### 2.3. Simulation of the Performance of a Full-Ring Small Animal PET Scanner Consisting of 20 Detectors

**Figure 5:**
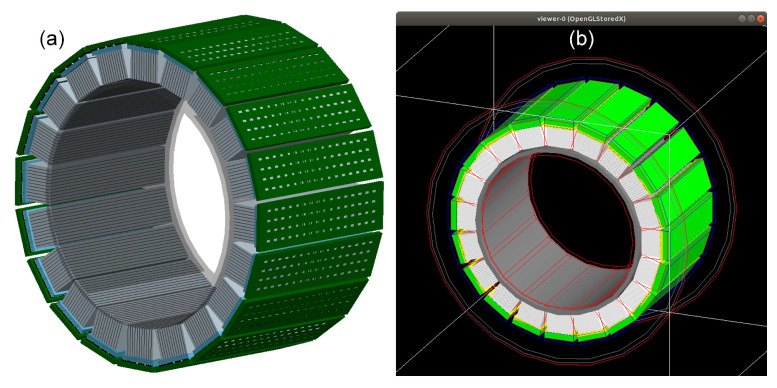
(**a**) Schematic and (**b**) GATE simulation views of the detector geometry of the full-ring small animal PET scanner consisting of 20 semi-monolithic detectors.

A full-ring PET scanner with 20 long semi-monolithic detectors (Figure 5) was simulated in this work. The scanner featured an 83 mm detector ring diameter and a 56 mm axial FOV. The gap between the front faces of adjacent detector modules was 0.73 mm. Monte Carlo simulations were performed for the full-ring PET scanner using the Geant4 Application for Tomographic Emission (GATE) software (v9.1) [42]. The physics list used in the simulation was QGSP_BERT_EMV, and the random generator used JamesRandom. The energy resolution of the detector was set to 15% at 511 keV, and an energy window of 350–650 keV was used. The coincidence timing window was set to 6 ns. The delay offsets were set to 300 ns with the same coincidence timing window of 6 ns. The policy used for the coincidence processing was takeWinnerOfGoods. The detector dimensions in the simulation were identical to those of the experimental detectors. During data analysis, Gaussian noise with FWHMs of 1.7 mm and 2.1 mm was added to the coordinates in the monolithic and DOI directions, respectively, to simulate the position resolution of the experimental detectors. First, a uniform cylinder (60 mm in both diameter and length) was simulated to obtain the normalization data, and a total of 8.4 × 10^6^ counts were simulated. A direct normalization was applied during the image reconstruction. Second, a 10 kBq point source was placed at different axial positions along the central axis at 4 mm intervals to estimate the sensitivity of the PET scanner. The simulation time at each position was 50 s. System sensitivity was calculated as the ratio of coincidence counts at each position to the total number of positron decays from the source. Third, an ultra-micro hot rod phantom with rod diameters of 0.75, 1.0, 1.35, 1.7, 2.0, and 2.4 mm was simulated at the center of the FOV and at a 15 mm radial offset. Total coincidence events of 6.7 × 10^6^ and 7.1 × 10^6^ were generated for the phantom at the center and at the 15 mm radial offset, respectively. The images were reconstructed using the same OSEM algorithm with PSF modeling.

## 3. Results

### 3.1. Detector Calibration

**Figure 6:**
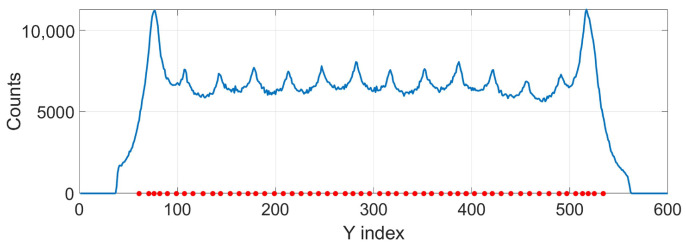
The y-profile of a slab obtained by uniform irradiation of the slab and the 55 boundaries (red dots) of the 56 y segments of 1 mm.

**Figure 7:**
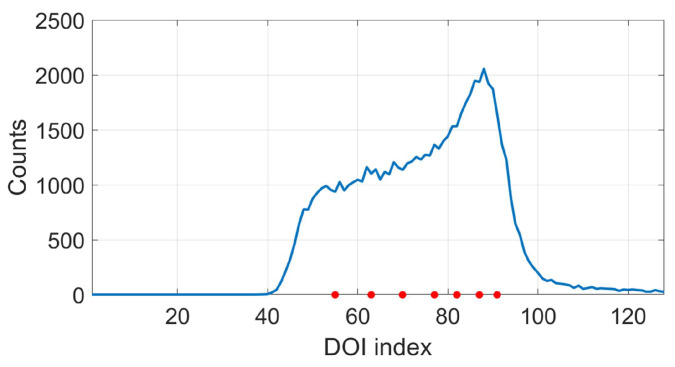
The DOI profile of a slab at center y position obtained by uniform irradiation of the slab and the 7 boundaries (red dots) of the 8 DOI segments of 1.25 mm.

The y-profile of a slab of the semi-monolithic detector using the ESR reflector and the 55 boundaries (red dots) of the 56 y-segments is shown in Figure 6. The y-profile was obtained using the squared COG method. The 55 boundaries were generated by dividing the area under the y profile into 56 equal areas. Due to the large gap (0.65 mm) between the SiPMs and the edge effect at both ends, the y-profile obtained from the ^176^Lu background was not uniform. The DOI profile of a slab of the ESR detector (Epic Crystal Co. Ltd., Shanghai, China) at the central y segment of 1 mm wide and the 7 boundaries (red dots) of the 8 DOI segments is shown in Figure 7. The DOI profile was obtained using the inverse standard deviation method, and the 7 DOI boundaries were generated using the same method as the y boundaries. In spite of the non-uniform distribution of both the y- and DOI profiles, a slab could still be easily divided into 56 different y segments of 1 mm and 8 different DOI segments of 1.25 mm with the proposed method. Any events happening in the slab could be assigned to different y and DOI segments using the y and DOI boundaries.

### 3.2. Spatial Resolution of the Prototype PET Scanner

**Figure 8:**
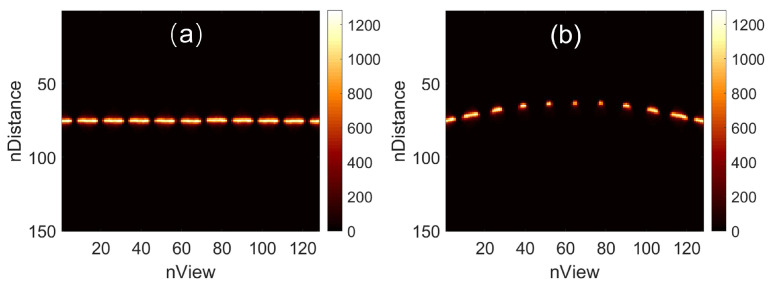
The summed sinograms of a ^22^Na point source placed at radial offsets of (**a**) 0 mm and (**b**) 5 mm.

**Figure 9:**
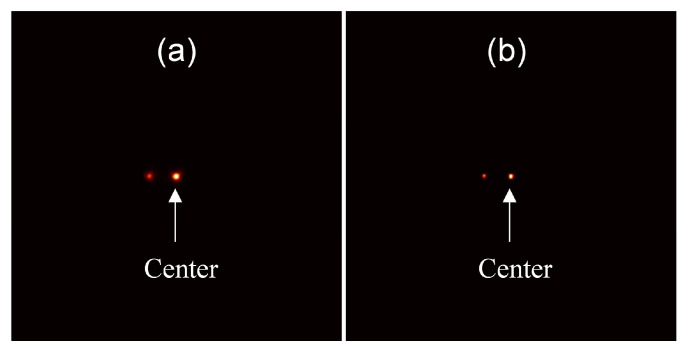
The images of a ^22^Na point source placed at radial offsets of 0 mm and 5 mm reconstructed (**a**) without PSF modeling, and (**b**) with PSF modeling.

**Figure 10:**
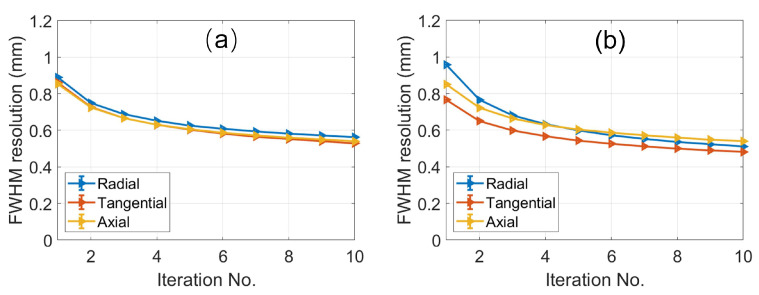
The spatial resolutions of the scanner as a function of the iteration number measured at radial offsets of (**a**) 0 mm and (**b**) 5 mm. The images were reconstructed with PSF modeling.

The sinograms summed over all planes of the ^22^Na point source placed at radial offsets of 0 mm and 5 mm are shown in Figure 8. The sinogram of the point source at a radial offset of 5 mm showed significant missing data due to the limited angle coverage. The reconstructed images of a point source placed at radial offsets of 0 mm and 5 mm are shown in Figure 9. The images were reconstructed using OSEM with 16 subsets and 5 iterations. The spatial resolutions of the scanner as a function of iteration number measured at both radial offsets with PSF modeling are shown in Figure 10, where error bars represent the root mean square error (RMSE) of the Gaussian fitting. As the number of iterations increases, the spatial resolution improves; however, the improvement plateaus after five iterations.

The spatial resolution results at different radial offsets are summarized in Table 1. The uncertainty indicates the root mean square error (RMSE) of the Gaussian fitting. The images were reconstructed using 5 iterations. The prototype scanner achieved radial and tangential spatial resolutions of ~1.1 mm and an axial spatial resolution of ~1.4 mm at both positions when the images were reconstructed without PSF modeling. Spatial resolutions of better than 0.65 mm across all three dimensions were obtained when PSF modeling was applied.

It should be noted that the PSF parameters used in the reconstruction were extracted from a point-source image acquired at the center of the FOV and reconstructed without PSF modelling. This represents an idealized scenario that does not account for the spatial variation of the PSF across the FOV, DOI effects at oblique incidence angles, or the radial dependence of the PSF kernel. Consequently, the sub-millimeter spatial resolution reported here with PSF modelling should be regarded as a best-case (upper-bound) estimate that may not reflect the resolution achievable for off-center or extended sources in a full-ring scanner.

### 3.3. Simulation Results of a Full-Ring PET Scanner

**Figure 11:**
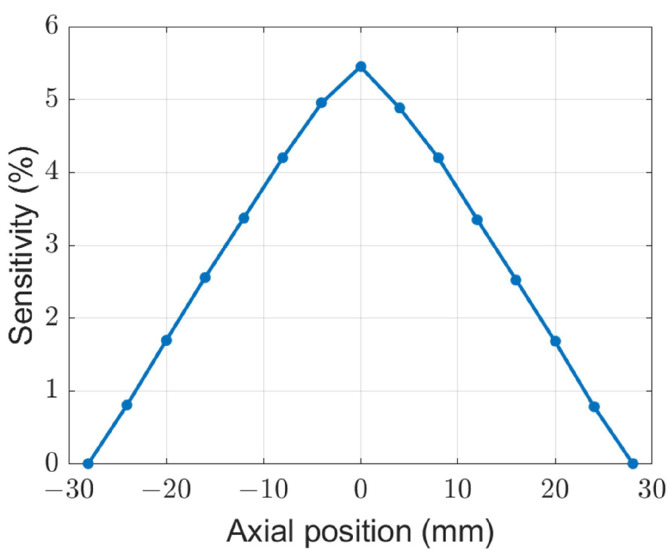
The simulated sensitivity of the full-ring PET scanner at different axial positions along the central axis.

Figure 11 shows the sensitivity of the full-ring PET scanner at different axial positions along the central axis. A peak sensitivity of 5.45% was achieved with an energy window of 350–650 keV.

**Figure 12:**
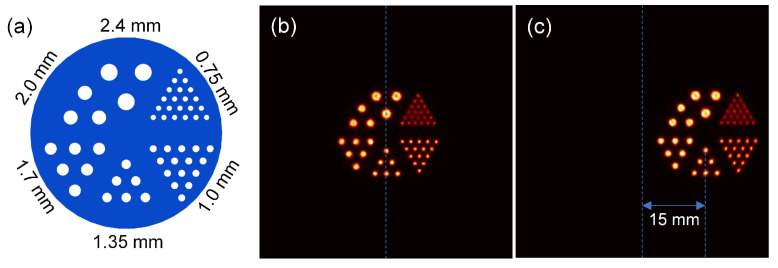
(**a**) Schematic of an ultra-micro hot rod phantom and transverse images reconstructed from simulation data: (**b**) phantom placed at the center and (**c**) phantom with a 15 mm radial offset. The number of iterations is 10.

Figure 12b,c show the transverse images of the ultra-micro hot rod phantom placed at the center and at a 15 mm radial offset, respectively, reconstructed using OSEM with PSF modeling (10 iterations). Rods as small as 0.75 mm were clearly resolved in both the center and the 15 mm radial offset positions. This demonstrates the system’s ability to maintain high spatial resolution even at the periphery of the FOV, thanks to the DOI capability of the detectors.

## 4. Discussion

The semi-monolithic scintillator detectors offer intrinsic depth-encoding capability as compared to the segmented crystal array detector, and exhibit reduced edge effects as compared to the monolithic scintillator detector. Furthermore, the edge effects can be further mitigated by increasing the slab length. Unlike segmented crystal array and monolithic scintillator detectors, which have been extensively studied and widely used in small animal PET scanners, semi-monolithic detectors have only been implemented in one small animal PET scanner to date. This work evaluated the initial performance of a prototype small animal PET scanner with long semi-monolithic detectors through experimental measurements and Monte Carlo simulations. Unlike the previous semi-monolithic PET scanner which used shorter slabs (25.6 mm [35]), our work utilized long semi-monolithic scintillator detectors (56 mm) to significantly reduce edge effects of the detector. A dedicated mouse imaging PET scanner could possibly be built with a single detector ring, which further reduces the degradation of the spatial resolution of the scanner results from the edge effects since the detector volumes affected by the edge effect are located at the edge of the axial FOV.

In the experimental measurements, the spatial resolution of a prototype small animal PET scanner consisting of only two semi-monolithic detectors and a rotation platform was measured. The spatial resolution was only measured at 0 mm and 5 mm radial offsets since the transverse FOV of the prototype scanner was only 12.4 mm. At the 5 mm radial offset, data gaps were evident in the sinogram due to the limited angle coverage. A calibration method for y- and DOI-direction responses using ^176^Lu background radiation in LYSO scintillators was proposed and evaluated. This method is applicable to system-level calibration and effectively corrects non-uniform position responses in both y- and DOI directions. The prototype small animal PET scanner achieved radial and tangential spatial resolutions of ~1.1 mm and an axial spatial resolution of ~1.4 mm when the images were reconstructed without PSF modeling which can be attributed to the detector intrinsic resolutions: a slab thickness of 0.96 mm, y-position resolution of ~1.7 mm, and DOI resolution of ~2.1 mm [27]. With PSF modeling, spatial resolutions in all three directions improved to ~0.65 mm. This high resolution is attributed to the small crystal width (0.96 mm) and the application of PSF modeling during image reconstruction, which effectively models all physical and geometric factors affecting the spatial resolution of a PET scanner.

In Monte Carlo simulations, sensitivity and imaging performance were evaluated for a full-ring small animal PET scanner with 20 semi-monolithic detectors. A peak sensitivity of 5.45% was achieved with an energy window of 350–650 keV. Rods as small as 0.75 mm in diameter were clearly resolved in images of an ultra-micro hot rod phantom at both central and 15 mm radial offset positions when reconstructed with PSF modeling. This demonstrates the system’s ability to maintain high resolution even at the periphery of the FOV, mainly due to the high DOI resolution of the detectors.

The previous small animal PET scanner based on semi-monolithic detectors (with slab size of 0.97 mm × 25.6 mm × 12 mm) achieved spatial resolutions of 0.81/0.86/2.43 mm (radial/tangential/axial) and 0.87/0.81/2.3 mm at the center and a radial offset of 5 mm, respectively [35]. In contrast, our two-detector prototype achieved spatial resolutions of 0.63/0.60/0.61 mm (radial/tangential/axial) and 0.60/0.54/0.60 mm at the center and a radial offset of 5 mm, respectively. The previous scanner achieved a peak sensitivity of 3.5% with an energy window of 358–664 keV. In contrast, our simulated full-ring scanner achieved a peak sensitivity of 5.45% with an energy window of 350–650 keV. The higher sensitivity of our simulated scanner was mainly due to the large solid angle coverage of the full-ring geometry. The high spatial resolution of our two-detector prototype was mainly due to the better detector resolution and the application of PSF modeling during reconstruction.

The spatial resolution values better than 0.65 mm obtained from the two-detector prototype using PSF modelling should be interpreted with caution. The PSF parameters were derived from a centrally located point source, representing a best-case scenario that does not account for the spatially variant nature of the PSF across the FOV. In a realistic full-ring scanner, the effective PSF would vary radially and axially due to DOI effects, crystal penetration, and Compton scattering in the detector matrix, as well as oblique photon incidence angles from off-axis sources. Therefore, the reported sub-millimetre resolution should be considered an upper-bound estimate rather than a guaranteed performance metric for all source locations. In particular, the axial spatial resolution needs to be further investigated once a full-ring scanner is constructed.

The main limitation of semi-monolithic detectors is that the timing and energy calibrations are more challenging than those for conventional detectors. The simulation results demonstrate the ideal performance of a full-ring scanner, whereas the imaging performance of an actual full-ring scanner is expected to be suboptimal. The sensitivity can be further improved by increasing slab length or using multiple detector rings. The position resolution of the detectors in the monolithic direction was poor, and can be further improved by reducing the gaps between the SiPM sensor channels and using advanced positioning algorithms such as machine learning. The position resolution of the detectors in the segmented direction can be further improved by using detectors with thinner slabs.

## 5. Conclusions

In this work, the initial performance of a small animal PET scanner consisting of long semi-monolithic scintillator detectors was evaluated using both experimental measurements and Monte Carlo simulations. Using experimental data from the two-detector prototype PET scanner, spatial resolution better than 0.65 mm was obtained with PSF modelling. In Monte Carlo simulations of a full-ring PET scanner consisting of 20 such detectors, a peak sensitivity of 5.45% was achieved, and rods with a diameter of 0.75 mm were clearly resolved in phantom images reconstructed with PSF modelling. Long semi-monolithic scintillator detectors represent a promising candidate for developing small animal PET systems designed to achieve sub-millimeter uniform spatial resolution, good sensitivity, and reduced cost.

## Figures and Tables

**Table 1:** Summary of the measured spatial resolutions of the prototype PET scanner.

Radial Offset (mm)		Radial (mm)	Tangential (mm)	Axial (mm)
0	Without PSF	1.14 ± 0.009	1.10 ± 0.012	1.40 ± 0.014
	With PSF	0.63 ± 0.001	0.60 ± 0.001	0.61 ± 0.002
5	Without PSF	1.08 ± 0.009	1.06 ± 0.004	1.35 ± 0.008
	With PSF	0.60 ± 0.001	0.54 ± 0.001	0.60 ± 0.001

## Data Availability

The data presented in this study are available on request from the corresponding author due to institutional and data format restrictions.

## Abbreviations

The following abbreviations are used in this manuscript:

PET	Positron Emission Tomography
DOI	Depth of Interaction
FOV	Field of View
LYSO	Lutetium Yttrium Oxyorthosilicate
SiPM	Silicon Photomultiplier
PMT	Photomultiplier Tube
PDE	Photon Detection Efficiency
MRI	Magnetic Resonance Imaging
CT	Computed Tomography
SPECT	Single Photon Emission Computed Tomography
ESR	Enhanced Specular Reflector
COG	Center of Gravity
LUT	Lookup Table
OSEM	Ordered-Subsets Expectation Maximization
PSF	Point Spread Function
FWHM	Full Width at Half Maximum
GATE	Geant4 Application for Tomographic Emission
RMSE	Root Mean Square Error
